# Recent Status of Low-Birth-Weight Infants in Japan

**DOI:** 10.7759/cureus.81008

**Published:** 2025-03-22

**Authors:** Shunji Suzuki, Atsuo Itakura, Jun Takeda, Naho Morisaki

**Affiliations:** 1 Obstetrics and Gynecology, Japan Association of Obstetricians and Gynecologists, Tokyo, JPN; 2 Department of Obstetrics and Gynecology, Juntendo University Faculty of Medicine, Tokyo, JPN; 3 Department of Social Medicine, National Center for Child Health and Development, Tokyo, JPN

**Keywords:** 39 weeks of gestation, current status, japan, low-birth-weight infant, neonatal birth weight, neonatal macrosomia

## Abstract

Objective

In this study, we examined the recent trends in neonatal birth weight in Japan with the precise gestational age distinctions of 39 weeks, which is the period internationally considered to be the most optimal for delivery based on the perinatal outcomes.

Materials and methods

Firstly, we calculated the frequency of low-birth-weight (LBW) infants beyond 22 weeks of gestation in Japan using the birth notifications from 2000, 2010, and 2020. Secondary, we analyzed the birth weight trends of the first singleton male and female infants born to primiparous women at 39 weeks of gestation.

Results

The frequency of LBW infants increased significantly in 2010 compared to 2000 (odds ratio 1.13, 95% confidence interval 1.12-1.14, p < 0.01); however, it decreased significantly in 2020 (odds ratio 0.952, 95% confidence interval 0.943-0.961, p < 0.01), although it did not reach the level in 2000 (p < 0.01). The average neonatal birth weight born to primiparous women at 39 weeks of gestation in 2010 was significantly lower than in 2000 (p < 0.01); however, in 2020 it was significantly higher than in 2000 although some differences were observed between the neonatal sexes (p < 0.01).

Conclusion

The frequency of LBW infants has been declining from 2010 to 2020 in Japan. In addition, the average neonatal birth weight born to primiparous women at 39 weeks of gestation in 2020 was significantly higher than in 2010.

## Introduction

A recent systematic review investigated an increase in neonatal birth weights at term over time, particularly when considering data since 1950 [1]. However, in Japan, birth weight has been reported to be declining consistently from 1962 to 2004 [2-6]. In addition, the frequency of low-birth-weight (LBW) infants was higher in Japan than the average among the Organization for Economic Cooperation and Development (OECD) countries [7,8]. The high incidence of LBW infants in Japan has been suspected to be associated with the high frequency of pre-pregnancy underweight mothers and poor weight gain during pregnancy [8-11]. In Japan, the ideal used to be to ‘give birth small but raise a big baby', and most Japanese obstetricians had been opposed to relaxing the weight gain recommendations [11]. However, recently Japanese dieticians and public health groups have been sounding alarms over undernourished young women especially during pregnancy [6,10,11], because the impact of decreased neonatal birth weight might have the possibility of increasing disease burden among adults in Japan [11]. In addition, the findings have also suggested that environmental changes such as socioeconomic improvements influence prenatal growth. Based on the above review [1], a further study may be needed with precise gestational age distinctions to understand the trend and its effect on maternal and child health.

Therefore, the aim of the study was to clarify whether neonatal birth weight in Japan is still declining. In this study, we examined the recent trends in neonatal birth weight in Japan with the precise gestational age distinctions of 39 weeks.

## Materials and methods

The protocol for the study was approved by the Ethics Committee of the Japanese Red Cross Katsushika Maternity Hospital and the National Center for Child Health and Development.

In Japan, it is mandatory that the birth certificate, which is the official document from the Japanese Ministry of Health, Labor and Welfare, be submitted to the municipal office within 14 days of the birth [12]. Since 1991, the submission of the birth certificate has been required in principle for births after 22 weeks of gestation, as 22 weeks of gestation or later is currently considered premature delivery [13]. On the birth certificate, the neonatal sex, birth weight, height, gestational weeks of birth, and number of births to the mother are recorded.

Firstly, in this study we calculated the frequency of low-birth-weight infants beyond 22 weeks of gestation in Japan using the birth notifications from 2000, 2010, and 2020. In the examination, we could not examine the frequency of twin pregnancies because of the different ways in which single fetal demise is handled for each year.

Secondly, as a number of factors are involved in neonatal birth weight, we analyzed the birth weight trends of the first singleton male and female infants born to primiparous women at 39 weeks of gestation focusing on the period when perinatal outcomes are considered good. These inclusion criteria such as 39 weeks of gestation in the second analyses were selected for the reasons as follows. Since late 2012, the label ‘term’ has been replaced with the designations early term (37 0/7 weeks of gestation through 38 6/7 weeks of gestation), full term (39 0/7 weeks of gestation through 40 6/7 weeks of gestation) to more accurately describe deliveries occurring at or beyond 37 0/7 weeks of gestation according to the American College of Obstetricians and Gynecologists (ACOG) Committee Opinion [14]. In their discussion, the frequency of adverse neonatal outcomes was lowest among uncomplicated pregnancies delivered between 39 0/7 weeks of gestation and 40 6/7 weeks of gestation (= full term). In their discussion, the frequency of adverse neonatal outcomes was lowest among uncomplicated pregnancies delivered between 39 0/7 weeks of gestation and 40 6/7 weeks of gestation (= full term) [15,16]. Based on a previous large study in Japan, the mean ± standard deviation (SD) duration of pregnancy was 39.6 ± 1.6 weeks and the optimal gestational timing of delivery based on neonatal mortality was 39 weeks for singleton pregnancies [17]. In addition, elective induction of labor at 39 weeks compared with expectant management beyond that gestational age has been observed to be associated with a significantly lower risk of cesarean delivery, maternal peripartum infection, and perinatal adverse outcomes [18,19].

The birth weight of multiple pregnancies has been reported to be significantly lighter than that of singleton pregnancies born at the same weeks of gestation [20-22]. In addition, it has been well known that infants born to multiparous women have heavier birth weights than those born to primiparous women. In addition, male infants are heavier than female infants born under the same circumstances [23,24].

In this study, we also examined the frequency of macrosomia (neonate with a birth weight of 4,000 g or more without malformations or other gross abnormalities) [25,26], because macrosomia has been reported to be associated with excessive maternal weight gain [27].

Data are presented as mean ± SD or numbers (percentages, %). As the sample size is more than two groups with no correspondence, differences between the neonatal birth weight and frequency of LBW infant and macrosomia at each year were analyzed using unpaired t-test, chi-square test, and one-way analysis of variance after the normality assumptions were checked with the statistical software SAS version 8.02 (SAS Institute, Cary, NC, USA). Differences with p < 0.05 were considered significant.

## Results

Table 1 shows the changes in frequency of LBW infants in Japan from 2000, 2010, and 2020. The frequency of LBW infants increased significantly in 2010 compared to 2000 (odds ratio 1.13, 95% confidence interval 1.12-1.14, p < 0.01); however, it decreased significantly in 2020 (odds ratio 0.952, 95% confidence interval 0.943-0.961, p < 0.01), although it did not reach the level in 2000 (p < 0.01). The frequency of macrosomia decreased significantly in 2010 compared to 2000 (p < 0.01); however, it did not change significantly in 2020 (p = 0.48).

**Table 1:** Changes in frequency of low-birth-weight (LBW) infants in Japan from 2000, 2010, and 2020. Data are presented as number (percentage) †Chi-square test (p-values of <0.05 were considered significant). *vs. 2000, #vs. 2010

Year	Total number	Total number without missing data	Low-birth-weight infants	Chi-square value†	P-value†	Macrosomia	Chi-square value†	P-value†
2000	1217246	1206551 (99.1)	103861 (8.6)	-		13424 (1.1)	-	-
2010	1101634	1087645 (98.7)	104284 (9.6)	665.9*	< 0.01*	9358 (0.9)	370.1*	< 0.01*
2020	872448	861798 (98.8)	79043 (9.1)	198.2* & 97.8#	< 0.01*#	7496 (0.9)	296.0* & 0.498#	< 0.01* & 0.48#

Table 2 shows the birth weight trends of the first singleton male and female infants born to primiparous women at 39 weeks of gestation in Japan in 2000, 2010, and 2020. The average age of primiparous mothers for both male and female singleton infants at 39 weeks of gestation increased significantly from 29.2 ± 4.5 in 2000 to 30.7 ± 5.1 in 2010 and 31.5 ± 5.2 years in 2020 (F-value: 38.45 and 40.12, p < 0.01 by one-way analysis of variance). The average neonatal birth weight born to primiparous women at 39 weeks of gestation in 2010 was significantly lower than in 2000 (p < 0.01); however, in 2020 it was significantly higher than in 2000 although some differences were observed between the neonatal sexes (p < 0.01). The frequency of macrosomia decreased significantly in 2010 compared to 2000 (p < 0.01); however, it increased significantly in 2020 compared to 2000 (p < 0.01).

**Table 2:** Birth weight of the first singleton male and female infants born to primiparous women at 39 weeks of gestation in Japan in 2000, 2010, and 2020. Data are presented as number (percentage) ‡Unpaired t test (p-values of <0.05 were considered significant). †Chi-square test (p-values of <0.05 were considered significant). *vs. 2000, #vs. 2010

Year	Total	Neonatal birth weight (g)	T-value‡	P-value‡	Low-birth-weight infants	Chi-square value†	P-value†	Macrosomia	Chi-square value†	P-value†
Male										
2000	92941	3121.2 ± 338.4	-	-	2884 (3.1)	-	-	493 (0.5)	-	-
2010	75569	3108.6 ± 340.4*	139.7*	< 0.01*	2351 (3.1)	0.009*	0.93*	267 (0.4)*	29.12*	< 0.01*
2020	62590	3130.6 ± 339.1*#	98.78* & 220.8#	< 0.01*#	1588 (2.5)*#	42.89* & 40.71#	< 0.01*#	600 (1.0)*#	98.27* & 201.1#	< 0.01*#
Female										
2000	85934	3014.6 ± 338.4	-	-	4631 (5.4)	-	-	240 (0.3)	-	-
2010	70042	2999.4 ± 332.4*	1623.0*	< 0.01*	3978 (5.7)*	21.89*	< 0.01*	124 (0.2)*	17.33*	< 0.01*
2020	58387	3016.6 ± 330.2*#	20.37* & 168.6#	< 0.01*#	2908 (5.0)*#	2.062* & 30.65#	< 0.01*#	225 (0.4)*#	12.18* & 50.99#	< 0.01*#

## Discussion

The results of the current study will indicate that the neonatal birth weight in Japan, which had been on a downward trend since 1962 to 2004 [2-6], has shown a tendency to recover over the past 10 years. Although we are relieved by the current results, we also understand that further observation and examination are required to see whether birth weight will actually recover to the ideal level as in other developed countries [1].

In the latter decade of the study period, the average birth weight of singleton neonates born at 39 weeks of gestation had increased significantly, and the level surpassing that of 20 years earlier. At the same time, the overall frequency of LBW infants was also decreasing, but the level did not reach that of 20 years ago. The current results may be due to a sense of urgency about the current dietary situation of Japanese pregnant women that had been raised even before that time [28-31] although the optimal weight gain during pregnancy for pregnant Japanese women was revised in 2023 [10]. On the other hand, the failure of the overall frequency of LBW infants to recover to the level in 2000 may be due to the increased high-risk pregnancies associated with the rising maternal age [31,32]. The resulting increase in the frequency of macrosomia may be undesirable [25,26]; however, it is perhaps a good thing that Japan is following the world trend of increasing birth weights [1,11]. The latest Japanese government survey also showed the percentage of underweight women in their 20s has dropped slightly since 2013 due to the sounding alarms over undernourished young women especially during pregnancy by the Japanese dieticians and public health groups [6,10,11]. Although the maternal weight gain during pregnancy cannot be examined in the current study, it would be desirable for the current trend to be more extensible in Japan. The impact of the decreased neonatal birth weight might have the possibility of an increased disease burden among adults associated with their longevity in Japan [11,33], because many lines of evidence, including epidemiologic data and data from extensive clinical and experimental studies, have indicated that early life events play a powerful role in influencing later susceptibility to certain chronic diseases [33,34]. It is now generally thought that these phenomena play a role in the pathogenesis of cardiovascular and metabolic disorders [33]. Therefore, we hope that the existence of the above revised guidelines of gestational weight gain will contribute to the continuation of the trend [10].

As one of several serious limitations in this study, birth certificates do not allow for any analyses concerning clinical backgrounds. The influence of premature delivery on the current results cannot be also ruled out for the present results; however, we cannot examine the spontaneous or artificial premature delivery. Although the study results will be general, the presence of potential bias between birth certificate holders cannot be also ruled out. Therefore, it is presumed that the mechanisms leading to the current trend will require further investigation at the medical facilities. We understand that in this study there are some other limitations besides that. The methodology of the current study was very simple; however, the arguments may need to facilitate reproducibility. The influence of missing data, which accounts for 1-2% of the total, cannot be ruled out in an analysis of slight changes like this study. ‘Unknown' on the birth certificate, which is the basis for missing data, has been recognized when a prenatal visit is not attended and so on, and seems to be associated with high-risk pregnancies affecting the frequency of LBW infants. The effect of the increased maternal age during the study period is also unknown, because the effect of maternal age on neonatal birth weight has varied from report to report. Recent research suggested that maternal age alone may not independently predict low birth weight, indicating that other, unobserved factors could confound this association [35]. On the other hand, one previous study in China reported that advanced maternal age increases the risk for heavier birth weight-related adverse outcomes [36]. In addition, significant advances in infertility treatment have been observed during this period [37,38]. However, since an increase in maternal age and infertility treatment generally lead to an increase in high-risk pregnancies related to LBW, the increased weight of infants born at 39 weeks of gestation under such circumstances may possibly suggest that conditions including maternal diet might have been improved. Therefore, further study is required to determine the mechanisms leading to the current trend in conjunction with the expected changes due to recommended weight gain during pregnancy.

## Conclusions

We examined the recent trends in neonatal birth weight in Japan over the past 20 years. The frequency of LBW infants increased significantly in 2010 compared to 2000; however, it decreased significantly in 2020. In addition, the average neonatal birth weight born to primiparous women at 39 weeks of gestation in 2020 was significantly lower than in 2000; however, in 2020 it was significantly higher than in 2000.

The increase in neonatal birth weight in Japan since 2010 may be associated with a change in Japanese attitudes associated with perinatal care. In addition, the perinatal management expected to improve more in Japan will be hoped to contribute to the continuation of the trend.
